# A small heat shock protein (SlHSP17.3) in tomato plays a positive role in salt stress

**DOI:** 10.3389/fpls.2024.1443625

**Published:** 2024-10-11

**Authors:** Guohua Cai, Mingyu Niu, Zhihao Sun, Huakun Wang, Shuo Zhang, Fei Liu, Yanqun Wu, Guodong Wang

**Affiliations:** School of Biological Sciences, Jining Medical University, Rizhao, Shandong, China

**Keywords:** salt stress, sHSP, SlHSP17.3, stress-related genes, transgenic Arabidopsis

## Abstract

Small heat shock proteins (sHSPs) are molecular chaperones that are widely present in plants and play a vital role in the response of plants to various environmental stimuli. This study employed transgenic *Arabidopsis* to investigate the impact of the new tomato (*Solanum lycopersicum*) sHSP protein (SlHSP17.3) on salt stress tolerance. Transient conversion analysis of *Arabidopsis* protoplasts revealed that SlHSP17.3 localized to the cytoplasm. Furthermore, as suggested by expression analysis, salt stress stimulated *SlHSP17.3* expression, suggesting that SlHSP17.3 is involved in the salt stress response of plants. *SlHSP17.3*-overexpressing plants presented greater germination rates, fresh weights, chlorophyll contents, and Fv/Fm ratios, as well as longer root lengths, lower reactive oxygen species (ROS) levels, and lighter cell membrane injury under salt stress. Furthermore, certain stress-related genes (*AtCOR15*, *AtDREB1B*, and *AtHSFA2*) were up-regulated in salt-stressed transgenic plants. Overall, *SlHSP17.3* overexpression improved the salt stress resistance of transgenic plants, mainly through increasing *AtCOR15*, *AtDREB1B*, and *AtHSFA2* expression.

## 1 Introduction

Salt stress is induced by elevated levels of soluble salts in the soil; it presents considerable challenges to plant growth and global agricultural productivity and constitutes a critical concern for global resources and ecology (Guo et al., 2022). Soil salinity, often exacerbated by irrigation practices and environmental factors such as arid climates and proximity to saltwater bodies, poses a threat to the sustainable cultivation of crops. Salt stress adversely affects various physio-biochemical events crucial for plant development.

Plants engage various complex adaptive mechanisms in response to salt stress to counteract harmful effects and preserve the cellular balance. This includes the activation of small heat shock proteins (sHSPs), the molecular chaperones instrumental for cellular responses to stress, which are especially useful in shielding cells against the negative impacts of thermal stress (Waters, 2013; Papsdorf and Richter, 2014; He et al., 2021). These proteins, typically ranging from 12–42 kDa, are recognized for their capacity to bind to and stabilize unfolded or partially folded proteins, thus preventing protein aggregation while assisting in later refolding (Sun et al., 2016). sHSPs, which are ancient and diverse, share one highly conserved α-crystallin domain (ACD), one variable N-terminal region, and one short C-terminal sequence. In most sHSPs, the ACD or HSP20 region includes nine highly conserved β-sheets (Hibshman et al., 2023). In accordance with their molecular weights, such HSPs can be classified into six categories: sHSPs, HSP40 (DnaJ family), HSP60, HSP70, HSP90, and HSP100 (Sun et al., 2012). sHSPs are currently recognized as the most extensively studied and largest HSP family (Basha et al., 2012). They are subclassified into cytosolic classes I (CI-type) and II (CII-type), mitochondria, chloroplasts, peroxisomes, and endoplasmic reticulum categories on the basis of sequence similarity and intracellular location (Siddique et al., 2008; Jiang et al., 2009). sHSP compartmentalization in plants not only highlights their role in maintaining cellular balance but also indicates functional specialization depending on their location, enhancing the overall stress resilience of plants.

*sHSP* expression within plants is induced by stresses such as heat, salt, cold, drought, heavy metals, and oxidation, underscoring their versatility and essential role in cellular defense mechanisms (Wu et al., 2022). These proteins are inducible, indicating a highly regulated transcriptional process, with heat shock factors (HSFs) activating the expression of HSPs, including sHSPs, upon stress (Wang et al., 2019a; Iqbal et al., 2022). This regulation plays a vital role in cell survival under unfavorable conditions, demonstrating the integral role of sHSPs in the cellular stress response. Research on plant sHSPs indicates that they are also involved in stress responses and development, not just functioning as molecular chaperones. For example, the expression of CI-type sHSPs increases during embryo formation and seed maturation (Wang et al., 2014). Additionally, sHSP proteins decrease light dependence during tomato seed germination (Koo et al., 2015) and protect specific cell types in plant embryo sacs under heat stress (Ambastha and Leshem, 2020a). Yang et al. (2022) reported that overexpression of *GmHSP17.9* in transgenic soybeans results in significant increases in fresh weight, nodule number, poly-β-hydroxybutyrate (PHB) content, nitrogenase activity, urea, and total nitrogen level, along with a notable increase in seed yield. Feng et al. (2019) reported that *CaHSP25.9* overexpression in *Arabidopsis thaliana* enhances the germination rate and length of roots under drought stress. Tomato SlHSP17.7 has been identified as a cofactor for SlCCX1-like, which targets endoplasmic reticulum (ER) membrane proteins to maintain intracellular Ca^2+^ homeostasis and reduce cold stress sensitivity (Zhang et al., 2020). As discovered by Qin et al. (2022), transgenic *Arabidopsis* plants overexpressing *TaHSP17.6* grow more lateral roots to adapt to salt stress. According to Tian et al. (2022), compared with their wild-type (WT) counterparts, transgenic tobacco plants harboring the *PtsHSP17.2* gene presented less variation in chlorophyll levels, malondialdehyde (MDA) levels, and relative electrolyte leakage upon heat stress. Hence, although the effects of stress-responsive *sHSP* genes have been documented, further extensive characterization of such abiotic stress responses is warranted to fully understand stress resistance in plants.

Numerous *sHSP* genes in plants respond to various signals; however, the specific roles of these genes in tomatoes remain unclear. This study isolated the stress-related *sHSP* gene *SlHSP17.3* from tomatoes and characterized it to elucidate its functions. The expression of *SlHSP17.3* can be triggered upon salt stress. Additionally, phylogenetic tree analysis together with subcellular localization studies revealed SlHSP17.3 as the CII-type sHSP. The overexpression of *SlHSP17.3* in *Arabidopsis* enhances salt stress resistance.

## 2 Materials and methods

### 2.1 Plants and treatments

Wide-type *Arabidopsis* (WT, ecotype Columbia-0, retained by our laboratory) and T_3_ transgenic *Arabidopsis* plants (genetic transformation obtained by our laboratory) were cultivated in quartz sand at 22°C (day)/20°C (night), with a 14-h/10-h light/dark cycle and 200 μmol m^-2^s^-1^ photon flux density. The WT tomato variety (*S. lycopersicum* cv. L-402, retained by our laboratory) was cultivated in quartz sand at 25°C (day)/22°C (night), with a 16-h/8-h light/dark cycle and a 200 μmol m^-2^s^-1^ photon flux density. Each plant material was subjected to irrigation via Hoagland’s nutrient solution once/weekly.

To investigate the *SlHSP17.3* expression profile upon salt treatment, Hoagland nutrient solution supplemented with 250 mM NaCl was added to the roots collected from six-week-old WT tomato plants, whereas Hoagland nutrient solution treatment alone was used for the control group. Moreover, 100 µM ABA was sprayed on plant leaves, while water was sprayed on control plant leaves. To analyze salt resistance, 200 mM NaCl solution was added to two-week-old WT and T_3_ transgenic *Arabidopsis* plants for a 14-day period.

### 2.2 Cloning and bioinformatics analysis of *SlHSP17.3*

The *SlHSP17.3* coding sequence (CDS) was subjected to amplification based on WT tomato leaf cDNA via polymerase chain reaction (PCR) with specific primers (5′-CCATGGATGGATTTGAGGTTGATGGGT-3′ (forward), 5′-CACGTGAGCAACTTTGACCTGAATGG-3′ (reverse)). Then, we ligated the PCR products into the pMD19-T vector (TaKaRa, Beijing, China) prior to sequencing. Multiple sequence alignment was conducted with DNAMAN version 6.0 software (Lynnon Biosoft, USA), and phylogenetic analysis of the SlHSP17.3 protein was performed via MEGA version 5.05 software (Sudhir Kumar, Temple University, USA). Additionally, we acquired the amino acid sequences of sHSP proteins from various plants from GenBank (https://www.ncbi.nlm.nih.gov/genbank/) for comparison. The accession numbers are as follows: AtHSP21 (*Arabidopsis thaliana*, NM_118906); AtHSP25.3 (DQ446875); NtHSP26 (*Nicotiana tabacum*, D88584); OsHSP26 (*Oryza sativa*, AB020973); PhHSP21 (*Petunia×hybrida*, X54103); PsHSP21 (*Pisum sativum*, X07187); SlHSP21 (*Solanum lycopersicum*, LEU66300); TaHSP26.6 (*Triticum aestivum*, AF097659); TtHSP26.8 (*Triticum turgidum* ssp. Dicoccon, AJ971372; ZmHSP (*Zea mays*, EU966283); ZmHSP26 (L28712); AtHSP17.6 (NM_121240); AtHSP17.7 (O81822); CpHSP17.7 (*Carica papaya*, AY242075); GmHSP17.9 (*Glycine max*, P05477); LpHSP17.4 (*Lycopersicon peruvianum*, AY608694); MsHSP17 (*Medicago sativa*, X98617); PdHSP17.5 (*Prunus dulcis*, AF159562); PsHSP17.1 (P19242); SlHSP17.6 (LEU72396); ZmHSP17.5 (EU970990); AtHSP22.0 (Q38806); GmHSP22 (X63198); PsHSP22.7 (M33898); AcHSP (*Ananas comosus*, AY098528); AtHSP17.4 (NM_114492); AtHSP17.6A (NM_104679); AtHSP17.8 (NM_100614); AtHSP18.2 (NM_125364); CfHSPI (*Capsicum frutescens*, AY284925); CpHSP17.5 (AY387588); CsHSP17.5 (*Castanea sativa*, AJ582679); CsHSP (*Camellia sinensis*, EU727315); FaHSP (*Fragaria×ananassa*, U63631); HaHSP17.6 (*Helianthus annuus*, X59701); HaHSP17.9 (AJ237596); HvHSP17 (*Hordeum vulgare* ssp. vulgare) (Y07844); HvHSP18 (X64561); LpHSP19.9 (AJ225047); LpHSP20 (AJ225048); LpHSP20.1 (AJ225046); MdHSP1 (*Malus×domestica*, AF161179); MdHSP17.5 (EU636239); MsHSP18.2 (X58711); NtHSP18 (X70688); OsHSP16.9 (X60820); OsHSP17.8 (X75616); OsHSP18 (FJ383169); OsHSP20 (EU325986); RcHSP17.8 (*Rosa chinensis*, EF053229); SlHSP17.7 (AF123255); SlHSP17.8 (AF123256); SlHSP20 (SLU59917); TtHSP16.9 (AM709752); VuHSP17.7 (*Vigna unguiculata*, EF514500); ZmHSP17.2 (X65725); AtHSP15.7 (DQ403190); GmHSP (B0M1A7); OsHSP16.0 (Q652V8); AtHSP23.5 (NM_124523); AtHSP23.6 (NM_118652); PsHSP22 (X86222); SlHSP (AB017134); and ZmHSP22 (AY758275).

### 2.3 SlHSP17.3 subcellular localization

The full-length *SlHSP17.3* CDS was amplified via the corresponding primers (5′-CTCGAGATGGATTTGAGGTTGATGGGT-3′ (forward), 5′-GGTACCGTAGCAACTTTGACCTGAATGG-3′ (reverse)). The amplified *SlHSP17.3* CDS was subsequently inserted into the vector pEZS-NL after XhoI/KpnI digestion, resulting in the generation of a *SlHSP17.3*::EGFP (enhanced green fluorescent protein) fusion construct regulated via the cauliflower mosaic virus (CaMV) 35S promoter. We subsequently transfected *Arabidopsis* mesophyll protoplasts with both EGFP and *SlHSP17.3*::EGFP recombinant plasmids, and fluorescence was observed using laser confocal microscopy (Leica TCS SP8, Leica, Wetzlar, Germany).

### 2.4 Transgenic *Arabidopsis* plant transformation and identification

We cloned the *SlHSP17.3* CDS in the CaMV 35S promoter-controlled pCAMBIA1302 binary expression vector and subsequently inserted this resulting recombinant plastid into *Agrobacterium tumefaciens* strain GV3101, which was then verified via PCR. *Arabidopsis* (Col-0) transformation was accomplished via the floral dip approach. After the transgenic plants were cultivated on half-strength MS agar plates supplemented with 25 µg/mL hygromycin for the identification of positive lines, DNA was extracted from WT plants and hygromycin-resistant T_1_ seedlings. The presence of target genes was confirmed by a PCR assay via the following primers: 35S promoter TACGCAGCAGGTCTCTCAAGACGAT (forward) and *SlHSP17.3* CACGTGAGCAACTTTGACCTGAATGG (reverse). Subsequently, ten separate homozygous transgenic T_3_ lines were obtained. To ensure uniform viability, all the seeds utilized in each assay were collected at identical stages and preserved under identical conditions.

### 2.5 Amplification of the *SlHSP17.3* promoter and GUS staining analysis

Using the cetyl-trimethylammonium bromide (CTAB) approach, the *SlHSP17.3* promoter region was amplified from genomic DNA isolated from leaf samples of WT tomato plants. This genomic DNA served as the template for amplification. For GUS staining analysis, T_3_ transgenic *Arabidopsis* seedlings expressing *SlHSP17.3*pro::GUS were subjected to both natural conditions and salt stress treatment. GUS staining was conducted following the methods of Sundaresan et al. (1995). The quantitative analysis of GUS staining was mainly based on the description by Ambastha and Leshem (2020b).

### 2.6 Salt stress assay

In the salt assay, we sprayed surface sterile T_3_ and WT progeny seeds on half-strength MS media supplemented with NaCl at varying concentrations (0, 100, and 150 mM); following two days of vernalization in the dark at 4°C, we placed the plates in the chamber and measured the germination rate daily thereafter. To analyze root growth after germination, we first sowed the seeds onto half-strength MS media for a three-day period and subsequently added the seeds with emerged radicles to half-strength MS media containing various NaCl concentrations (0, 100, and 150 mM) for fivedays. We subsequently assessed root elongation and fresh weight. For the germination experiments, under different salt concentrations, each genotype had 49 seeds per plate with three plates per treatment, resulting in 147 seeds per genotype per experiment. For the root length experiments, under different salt concentrations, each genotype had five seedlings per plate with three plates per treatment, for a total of 15 seedlings per genotype per experiment. To analyze salt resistance in mature plants, 200 mM NaCl was applied for continuous treatment of four-week-old WT and T_3_ adult plants over a two-week duration, after which the phenotype was observed. The chlorophyll contents were quantified according to the approach by Kong et al. (2014). Each experiment was conducted three times.

### 2.7 Chlorophyll fluorescence analysis

Chlorophyll fluorescence imaging was conducted using a FluorCam multispectral fluorescence imaging system (PSI, Norfolk, USA) following the procedures outlined by Baker (2008). Chlorophyll fluorescence was measured using the Handy PEA instrument (Hansatech Instruments, Norfolk, UK) in accordance with the method from Ma et al. (2013). We subsequently determined the Fv/Fm ratio of PSII using the following equation: Fv/Fm = (Fm-Fo)/Fm.

### 2.8 Histochemical analysis and H_2_O_2_ and O_2_^•−^ determinations

For hydrogen peroxide (H_2_O_2_) and superoxide radical (O_2_^•-^) staining, the leaves were immersed in 3,3’-diaminobenzidine (DAB) staining solution (DAB dissolved in 50 mM Tris-acetate buffer, pH 5.0, with a final concentration of 0.1 mg/mL) and nitro blue tetrazolium (NBT) staining solution (NBT dissolved in 25 mM phosphate buffer, pH 7.6, with a final concentration of 0.1 mg/mL), respectively. The leaves were incubated at room temperature in the dark overnight. Afterward, the leaves were removed and immersed in a bleaching solution (ethanol:acetic acid:glycerol at 4:1:1), boiled for 10 min for decolorization, and then photographed. Trypan blue staining was carried out as described by Choi et al. (2007). H_2_O_2_ and O_2_^•−^ levels in both transgenic and WT leaf samples were subsequently analyzed via the method from Kong et al. (2014). For H_2_O_2_ and O_2_^•-^ staining, 200 mM NaCl solution was added to four-week-old WT and T_3_ transgenic *Arabidopsis* plants for a two-day period. For trypan blue staining, 200 mM NaCl solution was added to four-week-old WT and T_3_ transgenic *Arabidopsis* plants for five days.

### 2.9 Physiological parameter measurements

For physiological parameter measurements, 200 mM NaCl solution was added to four-week-old WT and T_3_ transgenic *Arabidopsis* plants for two days. A total of 1.0 g of leaf tissue was rapidly ground in 5 mL of cold extraction buffer (50 mM potassium phosphate buffer, pH 7.0, containing 1 mM EDTA and 1% polyvinylpyrrolidone). After homogenization, the mixture was centrifuged at 12,000 ×g for 20 min at 4°C. The resulting supernatant was immediately used as the crude enzyme extract for assessing antioxidant enzyme activity. The determination of superoxide dismutase (SOD, EC 1.15.1.1) activity primarily followed the method of Giannopolitis and Ries (1977) with slight modifications. 0.1 mL aliquot of the crude enzyme solution (the control group using potassium phosphate buffer at 50 mM) was immediately added to the reaction mixture (1.5 mL of 50 mM potassium phosphate buffer, 0.3 mL of 65 mM methotrexate solution, 0.3 mL of 0.5 mM NBT solution, 0.3 mL of 0.1 mM EDTA-Na_2_, and 0.3 mL of 0.2 mM riboflavin) and placed in a glass tube. The control group was incubated in the dark, while the experimental group was exposed to light at 4000 lx for 30 minutes. Subsequently, the absorbance at 560 nm was measured. The determination of catalase (CAT, EC 1.11.1.6) activity primarily followed the method of Aebi (1984) with slight modifications. The following solutions in sequence (0.2 mL of crude enzyme extract, 1.5 mL of phosphate buffer at pH 7.8, and 1.0 mL of distilled water) were added in a 10 mL test tube. Then 0.3 mL of 0.1 mol L^-1^ H_2_O_2_ was added to the test tube, the timer started immediately, and the solution quickly transferred into a quartz cuvette. The absorbance at 240 nm was measured and a reading was taken every 1 minute for a total of 3 minutes. After the measurement, the enzyme activity was calculated according to the following formula: Catalase activity (U g^-1^ FW min^-1^) =△A_240_×V_T_/0.1×V×t×FW. V_T_ represented total volume of the crude enzyme extract (mL). 0.1 refers to one enzyme activity unit (U) for every 0.1 decrease in A_240_. V represented volume of crude enzyme used for the determination (mL). t represented time from adding hydrogen peroxide to the last reading (min). FW represented fresh weight of the sample (g). The MDA level and relative electrical conductivity (REC) of the leaves were subsequently analyzed via the methods described by Kong et al. (2014). The ABA content of the leaves was assessed as described by Wang et al. (2019b).

### 2.10 Real-time quantitative PCR assay

The extraction of total leaf RNA was performed according to the method provided with the MolPure^®^ Plant RNA Kit (Yeasen, Shanghai, China). Reverse transcription was carried out with the TaKaRa Reverse Transcription Kit to obtain cDNA. The relative gene expression was assessed via RT-qPCR, with cDNA used as the template and *EF-1α* (GenBank Accession No. LOC544055) and *AtUbiquitin* (At4g05320) as control genes. RT-qPCR conditions were 30 s at 95°C, 15 s at 95°C, 15 s at 55°C, and 15 s at 72°C for 42 cycles. Each treatment was repeated three times. **Supplementary Table S1** displays the RT-qPCR primers used. For the RT-qPCR assay, 200 mM NaCl solution was added to four-week-old WT and T_3_ transgenic *Arabidopsis* plants for a two-day period.

### 2.11 Statistical analysis

SigmaPlot version 12.5 (Systat Software, San Jose, CA, USA) and SPSS version 18.0 (Chicago, IL, USA) were used for statistical analysis. The results are presented as the means ± standard deviations of at least three replicates. Significance levels are shown as *p < 0.05 and **p < 0.01 compared with the control.

## 3 Results

### 3.1 Identification and bioinformatics analysis of *SlHSP17.3*

**Figure 1:**
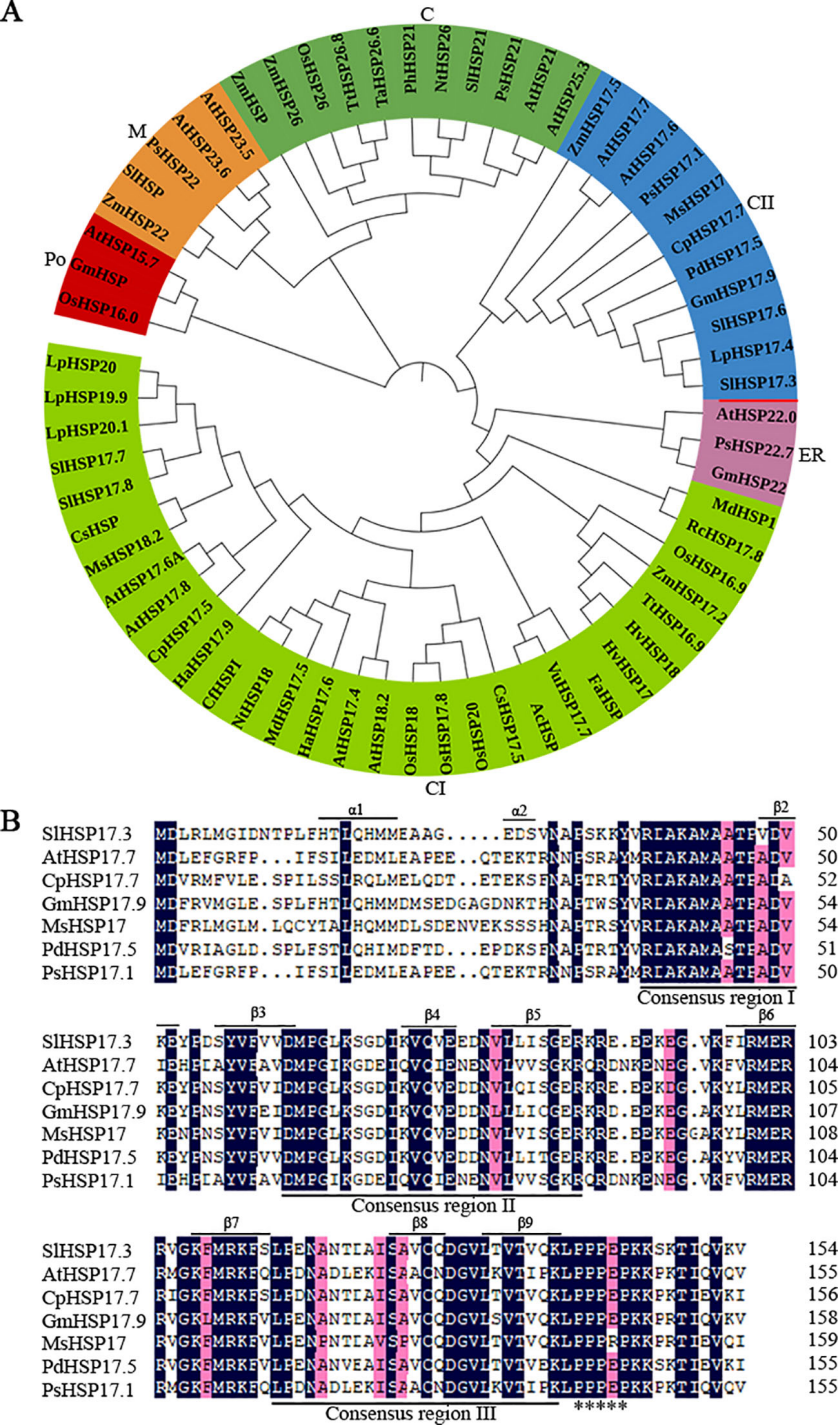
Phylogenetic tree and homologous sequence alignment analysis of sHSP proteins. **(A)** Phylogenetic tree analysis of *SlHSP17.3* with additional sHSP proteins, which divided the sHSP protein family into six clades (C, chloroplast; CII, cytosolic II; ER, endoplasmic reticulum; CI, cytosolic I; Po, peroxisome; M, mitochondria). The unrooted phylogenetic tree of sHSP proteins was generated using the neighbor-joining approach in MEGA (version 5.05). The accession numbers are as follows: AtHSP21 (*Arabidopsis thaliana*, NM_118906); AtHSP25.3 (DQ446875); NtHSP26 (*Nicotiana tabacum*, D88584); OsHSP26 (*Oryza sativa*, AB020973); PhHSP21 (*Petunia×hybrida*, X54103); PsHSP21 (*Pisum sativum*, X07187); SlHSP21 (*Solanum lycopersicum*, LEU66300); TaHSP26.6 (*Triticum aestivum*, AF097659); TtHSP26.8 (*Triticum turgidum* ssp. Dicoccon, AJ971372; ZmHSP (*Zea mays*, EU966283); ZmHSP26 (L28712); AtHSP17.6 (NM_121240); AtHSP17.7 (O81822); CpHSP17.7 (*Carica papaya*, AY242075); GmHSP17.9 (*Glycine max*, P05477); LpHSP17.4 (*Lycopersicon peruvianum*, AY608694); MsHSP17 (*Medicago sativa*, X98617); PdHSP17.5 (*Prunus dulcis*, AF159562); PsHSP17.1 (P19242); SlHSP17.6 (LEU72396); ZmHSP17.5 (EU970990); AtHSP22.0 (Q38806); GmHSP22 (X63198); PsHSP22.7 (M33898); AcHSP (*Ananas comosus*, AY098528); AtHSP17.4 (NM_114492); AtHSP17.6A (NM_104679); AtHSP17.8 (NM_100614); AtHSP18.2 (NM_125364); CfHSPI (*Capsicum frutescens*, AY284925); CpHSP17.5 (AY387588); CsHSP17.5 (*Castanea sativa*, AJ582679); CsHSP (*Camellia sinensis*, EU727315); FaHSP (*Fragaria×ananassa*, U63631); HaHSP17.6 (*Helianthus annuus*, X59701); HaHSP17.9 (AJ237596); HvHSP17 (*Hordeum vulgare* ssp. vulgare) (Y07844); HvHSP18 (X64561); LpHSP19.9 (AJ225047); LpHSP20 (AJ225048); LpHSP20.1 (AJ225046); MdHSP1 (*Malus×domestica*, AF161179); MdHSP17.5 (EU636239); MsHSP18.2 (X58711); NtHSP18 (X70688); OsHSP16.9 (X60820); OsHSP17.8 (X75616); OsHSP18 (FJ383169); OsHSP20 (EU325986); RcHSP17.8 (*Rosa chinensis*, EF053229); SlHSP17.7 (AF123255); SlHSP17.8 (AF123256); SlHSP20 (SLU59917); TtHSP16.9 (AM709752); VuHSP17.7 (*Vigna unguiculata*, EF514500); ZmHSP17.2 (X65725); AtHSP15.7 (DQ403190); GmHSP (B0M1A7); OsHSP16.0 (Q652V8); AtHSP23.5 (NM_124523); AtHSP23.6 (NM_118652); PsHSP22 (X86222); SlHSP (AB017134); and ZmHSP22 (AY758275). **(B)** Multiple sequence alignment of SlHSP17.3 with additional sHSP proteins. Conserved motifs are underlined. The asterisk indicates one polyproline motif. Secondary structure prediction shows α-helix at the N-terminus and β-sheets in the C-terminal ACD (β2-β9) underlined.

The *SlHSP17.3* gene is 773 base pairs (bp) long, and its open reading frame (ORF) is 468 bp. Its start codon (ATG) is located at the 148th nucleotide position, whereas the stop codon (TAG) is located at the 613th nucleotide position. The ORF is responsible for encoding one protein comprising 155 amino acids, and the estimated molecular weight is 17.3 kDa, whereas the isoelectric point is 6.75 (http://web.expasy.org/compute_pi/). According to a BLAST search of the tomato database (https://solgenomics.net/), this gene is located on chromosome 8 of the tomato genome. Phylogenetic tree analysis of reported sHSP proteins revealed that SlHSP17.3 is a member of the cytosolic class II sHSP protein group (**Figure 1A**). The cytosolic class II sHSP sequences revealed a distinctive N-terminal conserved domain (RDAKAMAATPADV) (**Figure 1B**). Moreover, the conserved C-terminal domain known as the ACD, comprising about 90 amino acids and containing consensus regions II/III, was identified. In addition, a polyproline motif (PPPEP) was detected in the C-terminus (Bondino et al., 2012; Li et al., 2016).

### 3.2 Subcellular localization of SlHSP17.3

**Figure 2:**
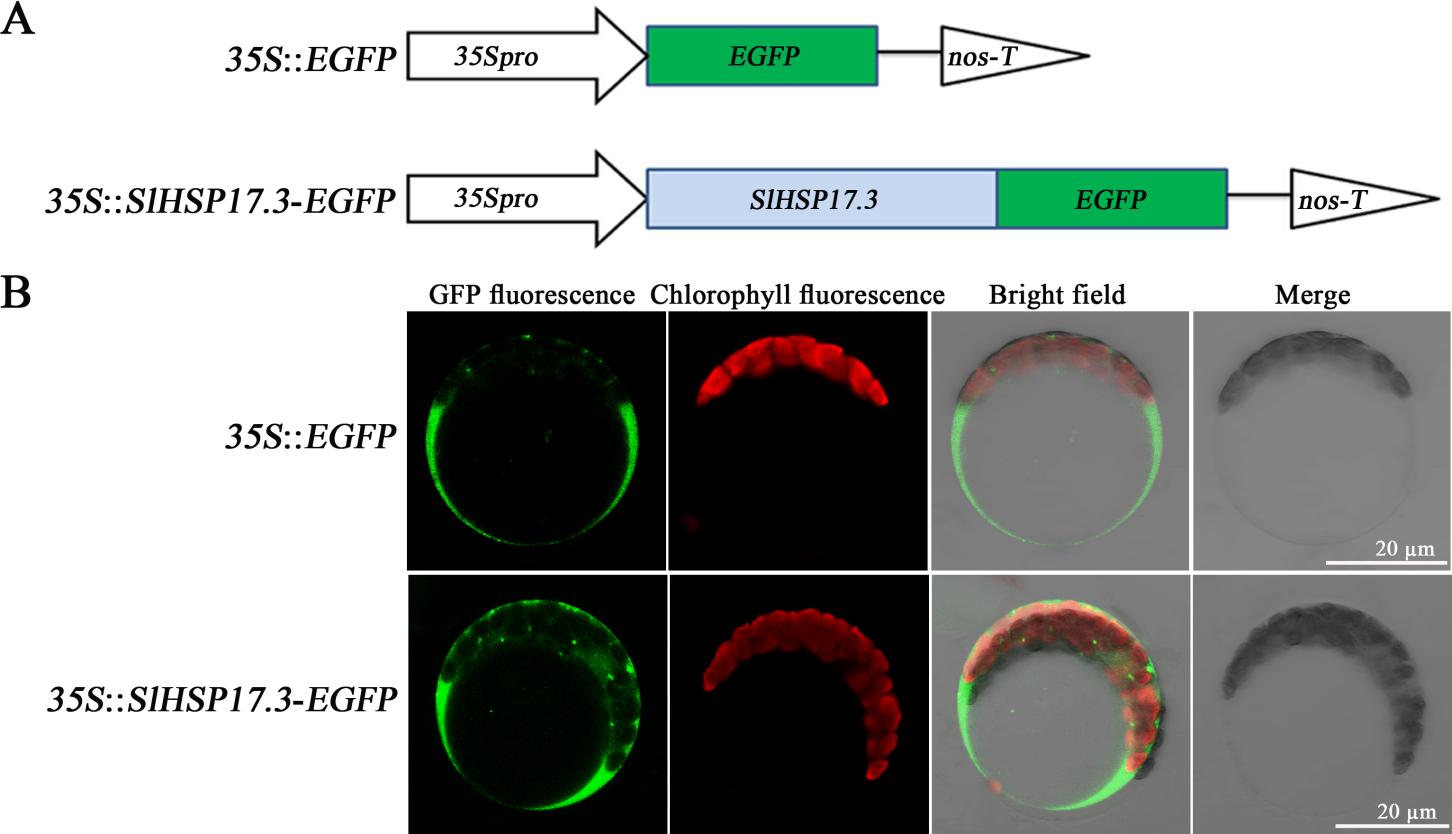
Subcellular localization of SlHSP17.3. **(A)** Structural pattern of the EGFP fusion protein. **(B)** 35S::EGFP (upper panel) and 35S::*SlHSP17.3*-EGFP (lower panel) transient expression in *Arabidopsis* protoplasts. Protoplasts were observed using laser confocal microscopy.

According to the ProtComp 9.0 database (http://www.softberry.com/berry.phtml) prediction, SlHSP17.3 is a potential cytoplasmic protein (**Supplementary Table S2**). For validation, transient transformation *in vivo* was conducted with *Arabidopsis* protoplasts isolated from leaves expressing 35S::EGFP and 35S::*SlHSP17.3*-EGFP fusion proteins (**Figure 2A**). 35S::EGFP exhibited dispersed green fluorescence throughout the protoplasts, with the exception of vacuoles (upper panels in **Figure 2B**). Conversely, transfection with the 35S::*SlHSP17.3*-EGFP fusion protein resulted in a clear concentration of green fluorescence within the cytoplasm (**Figure 2B**, lower panels). The results strongly support that SlHSP17.3 is a cytoplasmic protein.

### 3.3 Expression of *SlHSP17.3* induced by salt stress

**Figure 3:**
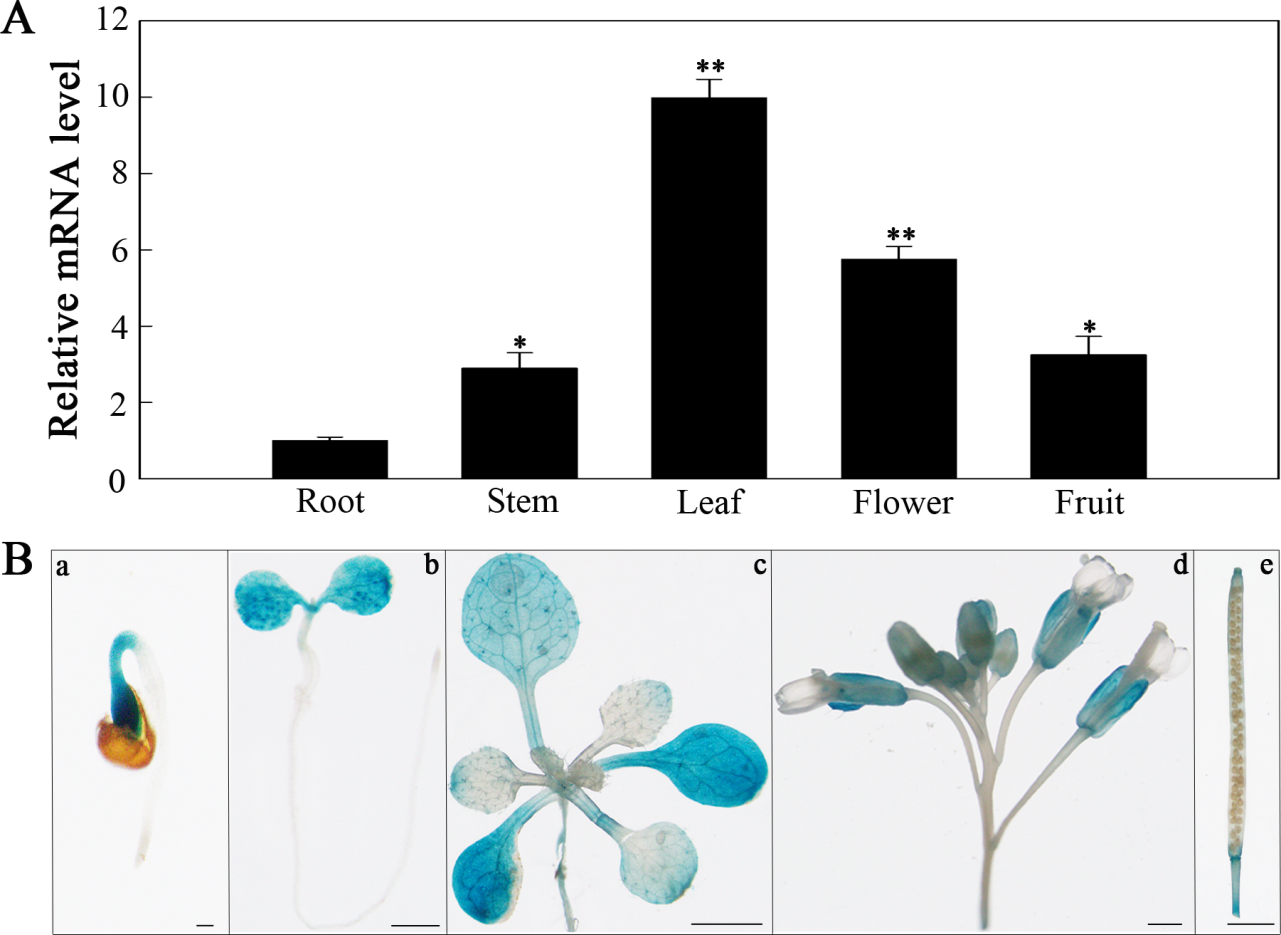
Tissue-specific expression analysis of *SlHSP17.3*. **(A)** RT-qPCR analysis of *SlHSP17.3* expression patterns in tomato. Significance levels are shown to be * for *p* < 0.05 and ** for *p* < 0.01. **(B)** GUS staining analysis of *SlHSP17.3*pro::GUS in different tissues of transgenic *Arabidopsis* plants grown under natural conditions. a, 1 day (bars = 200 µm); b, 3 days (bars = 1 mm); c, 12 days (bars = 1 mm); d, inflorescence (bars = 100 µm); e, silique (bars = 1 mm).

**Figure 4:**
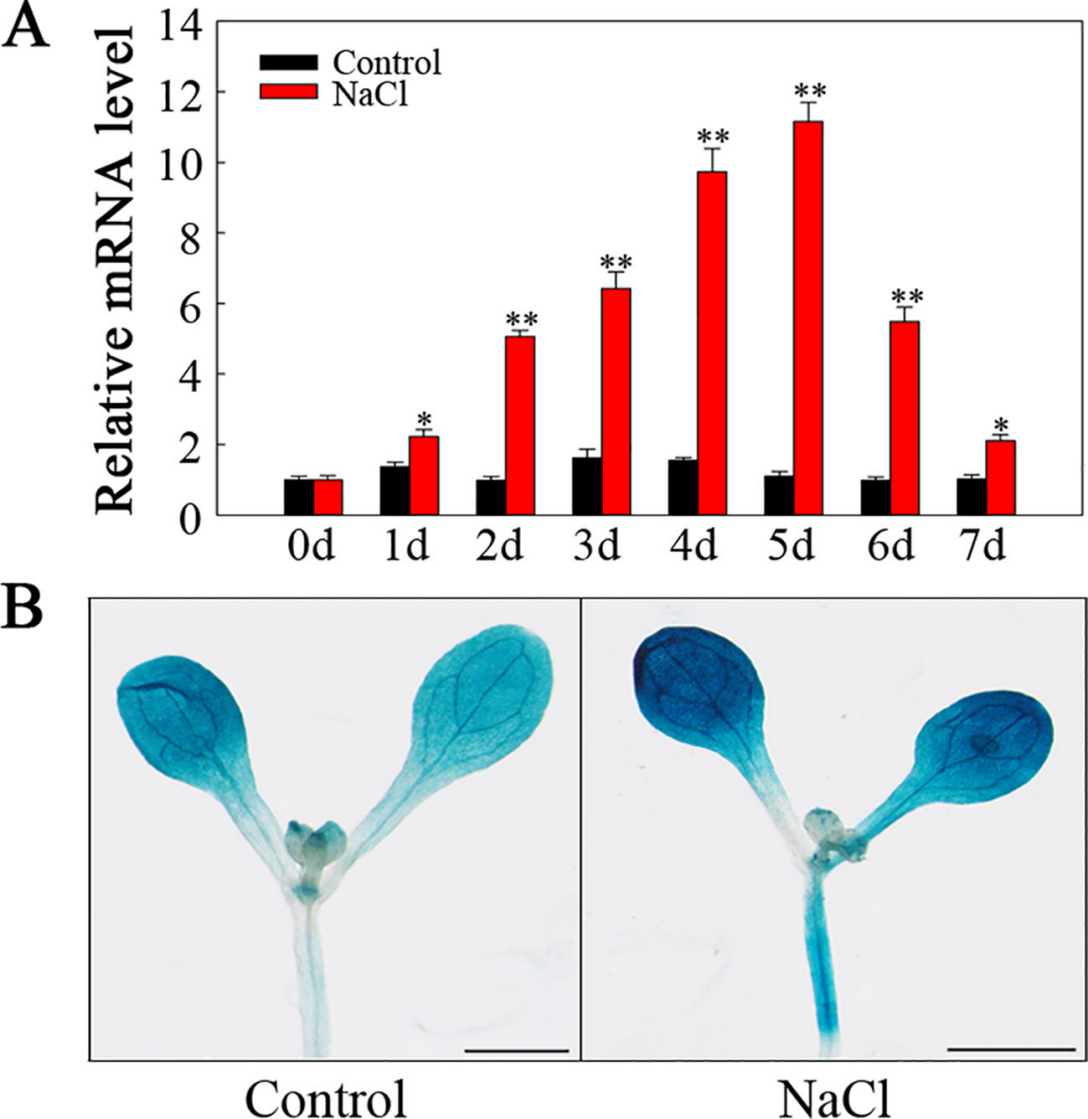
*SlHSP17.3* expression levels and GUS staining upon salt stress. **(A)** *SlHSP17.3* expression under salt stress according to RT-qPCR. **(B)** GUS staining analysis of seven-day-old leaves under normal conditions and after exposure to salt stress for one day. Scale bars = 1 mm. Significance levels are shown to be * for *p* < 0.05 and ** for *p* < 0.01.

To explore *SlHSP17.3* tissue-specific expression, two methods were employed. First, we conducted RT-qPCR to analyze *SlHSP17.3* expression patterns in various tomato organs. As shown in **Figure 3A**, *SlHSP17.3* exhibited consistent expression across different organs, with a preference for expression in the leaves. Second, we used GUS staining to examine *SlHSP17.3* expression in diverse tissues (**Figure 3B**). The results of the GUS staining analysis aligned with the RT-qPCR findings. Following treatment with 200 mM NaCl, the *SlHSP17.3* transcription level first increased but then decreased, peaking on day 5 (**Figure 4A**). Additionally, GUS staining revealed that the color intensity of the cotyledonary leaves and stems of three-day-old seedlings under salt stress was darker than that of untreated seedlings (**Supplementary Figure S1**). The same trend was observed for the cotyledonary leaves and stems of seven-day-old seedlings; although there was little difference in color intensity among the true leaves of seven-day-old seedlings, quantitative analysis of GUS staining also supported this result (**Figure 4B**; **Supplementary Figure S2**). We speculated that the expression of *SlHSP17.3* might be induced more prominently as the leaves mature. In summary, these experimental results indicate that the expression of *SlHSP17.3* is significantly upregulated under salt stress.

### 3.4 Screening of transgenic plants

**Figure 5:**
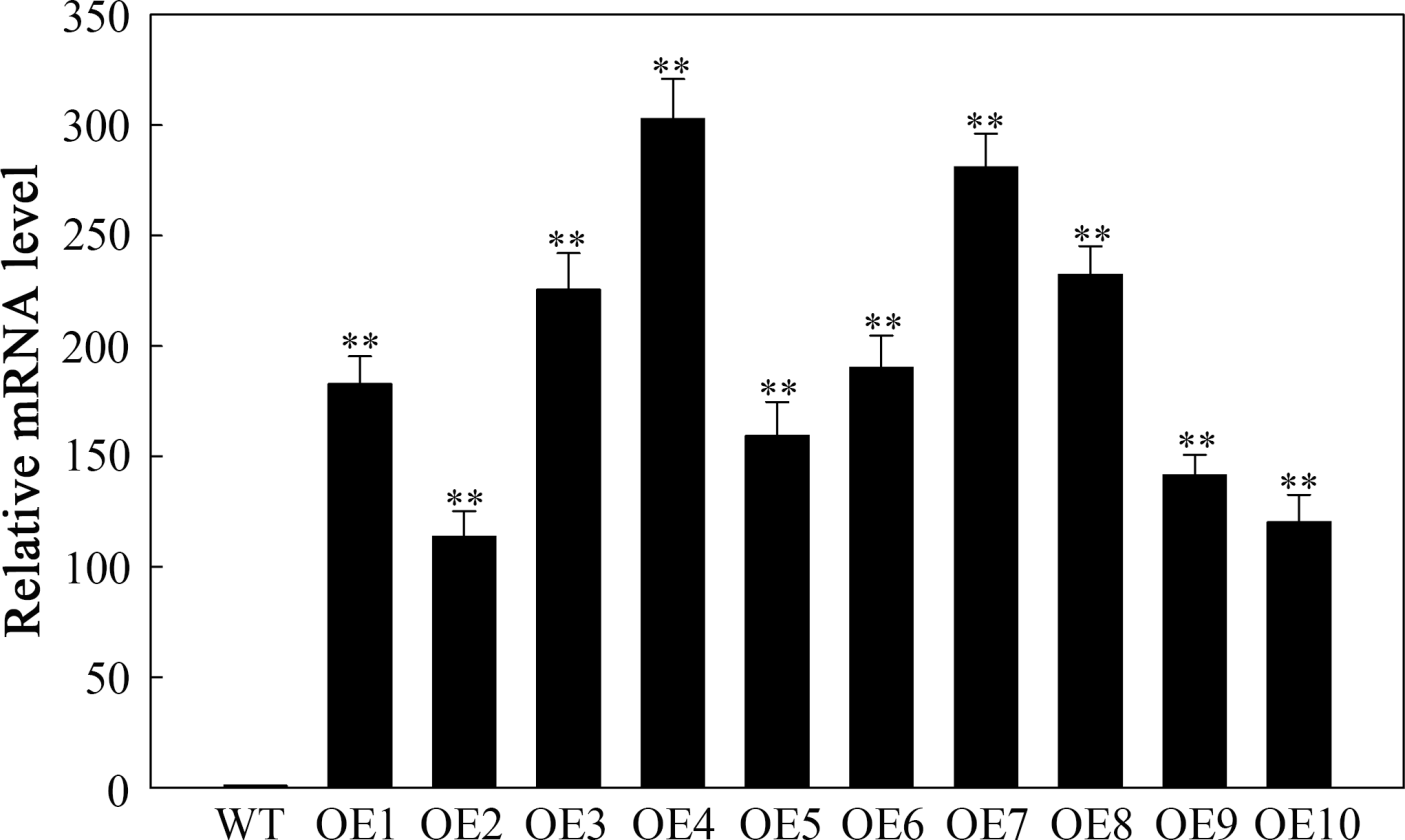
RT-qPCR detection of *SlHSP17.3*-overexpressing plants under natural conditions. Four-week-old WT and T_3_ generation transgenic *Arabidopsis* plants were used to detect the expression level of *SlHSP17.3*. Significance levels are shown as ** for *p* < 0.01.

Ten T_3_ transgenic lines resistant to hygromycin were evaluated using RT-qPCR. Compared with the WT, all ten transgenic lines presented increased expression of *SlHSP17.3* (**Figure 5**). From these lines, three were selected on the basis of their varying levels of relative expression: OE2 with low expression (113.6-fold), OE3 with moderate expression (225.6-fold), and OE4 with high expression (302.8-fold). These lines were selected for further experimentation.

### 3.5 *SlHSP17.3* overexpression improves salt tolerance

**Figure 6:**
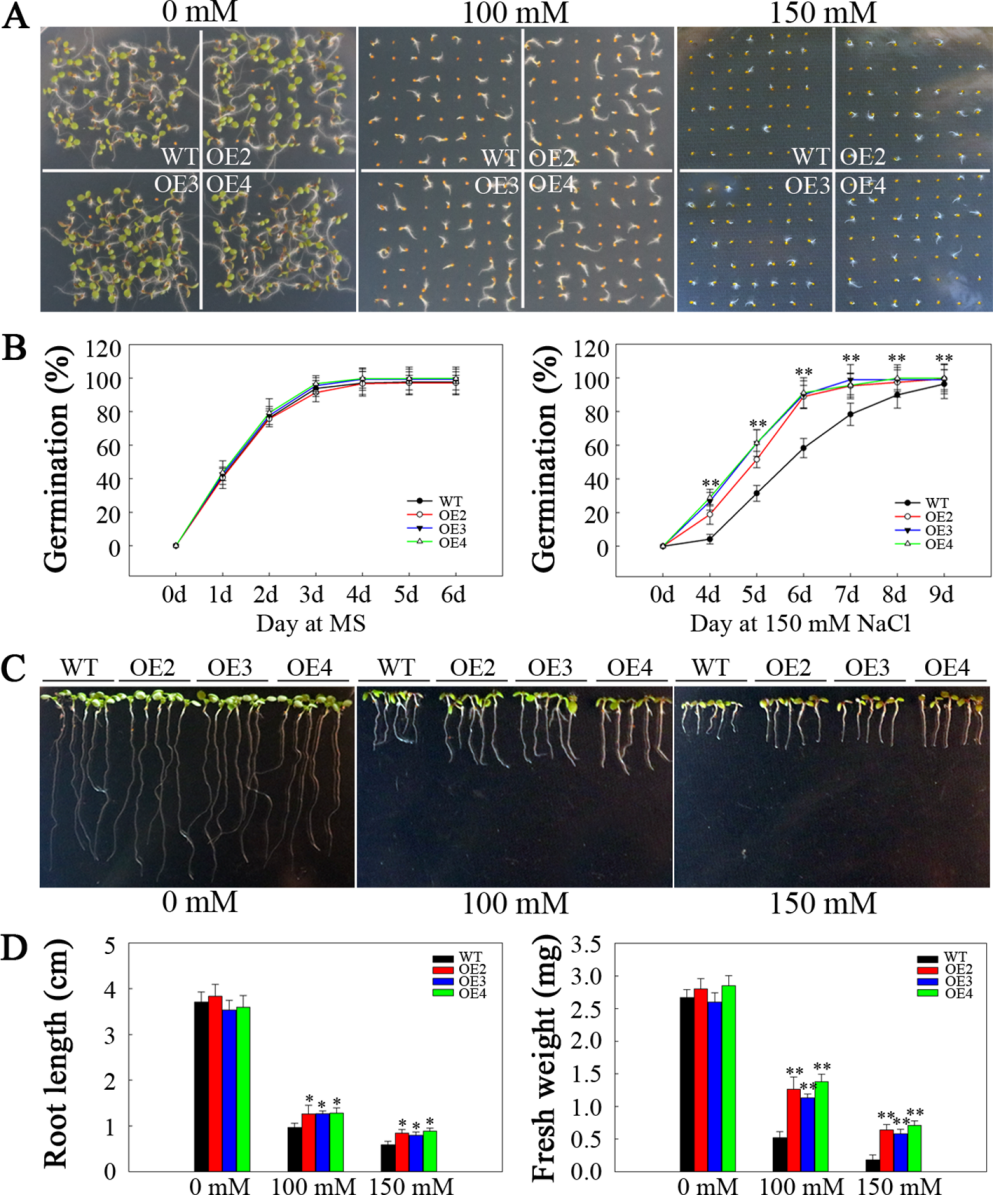
Analysis of the germination and seedling emergence of salt-stressed *SlHSP17.3*-overexpressing plants. **(A)** Seed germination under NaCl stress and normal conditions. **(B)** Germination rates of transgenic and WT plants under 150 mM NaCl stress and under normal conditions. **(C)** Seedling root phenotypes under NaCl stress and normal conditions. After sowing onto half-strength MS media for a three-day period, the seeds that had emerged from the radicle were placed onto half-strength MS media that contained varying concentrations of NaCl. The roots were photographed five days after transfer. **(D)** Seedling root length and fresh weight were measured five days after the plants were transferred to NaCl-containing plates. n = 147 per treatment for **(A)** and n = 15 per treatment for **(C)**. Significance levels are shown to be * for *p* < 0.05 and ** for *p* < 0.01.

To assess the potential role of SlHSP17.3 in the response of transgenic *Arabidopsis* plants to salt stress, we sowed seeds from both the transgenic and WT plants onto half-strength MS media supplemented with varying concentrations of NaCl. The germination rate did not markedly differ between the transgenic and WT plants on half-strength MS plates devoid of NaCl. Nonetheless, compared with WT plants, transgenic plants overexpressing *SlHSP17.3* presented significantly greater germination rates under 100 and 150 mM NaCl conditions (**Figures 6A, B**). Additionally, a seedling growth assay was performed under salt stress. The control plants presented a phenotype consistent with that of all the transgenic plants, whereas the WT plants presented a decreased cotyledon size, shortened root length, and lower fresh weight under salt stress (**Figures 6C, D**).

**Figure 7:**
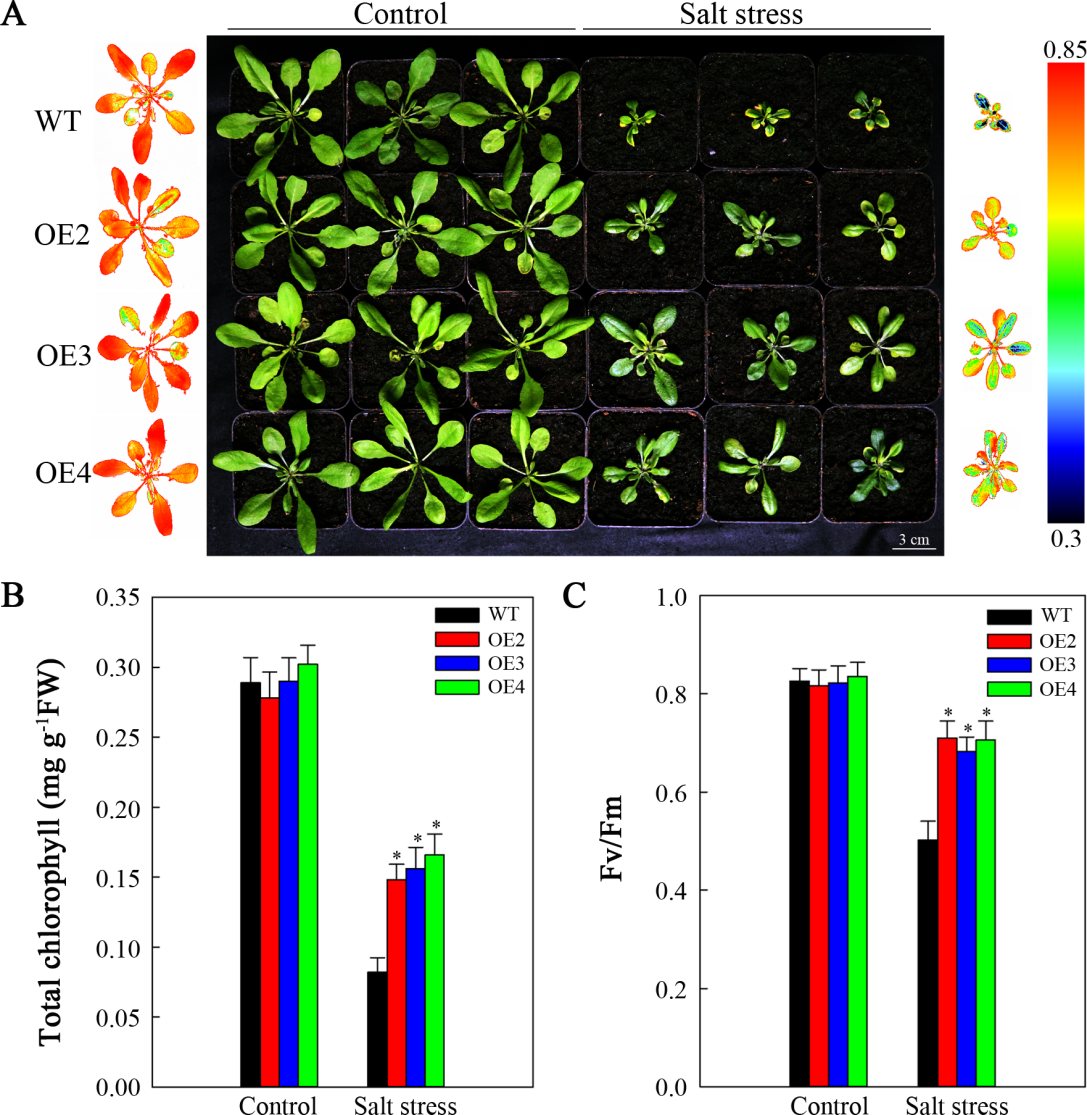
Analysis of the growth of mature salt-stressed *SlHSP17.3*-overexpressing plants. **(A)** Four-week-old WT and transgenic plants under 200 mM NaCl stress and normal conditions for two weeks. The chlorophyll fluorescence images on the left represent the leftmost column of plants in the control group, whereas the chlorophyll fluorescence images on the right represent the rightmost column of plants in the treatment group. **(B)** Total chlorophyll content. **(C)** Fv/Fm. Significance levels are shown as * for *p* < 0.05.

This study aimed to determine whether the improved salt resistance resulting from *SlHSP17.3* overexpression extends to mature plants. For this purpose, two-week-old transgenic and WT lines were subjected to 200 mM NaCl treatment for two weeks. As shown in **Figure 7A**, the control lines exhibited healthy growth with no discernible differences in growth phenotype. However, following salt stress, both the transgenic and WT plants presented obvious growth inhibition to differing extents. Nevertheless, the degree of inhibition in the WT plants was notably greater than that in the transgenic plants. Compared with the transgenic plants, the WT plants presented significantly smaller leaves, lower chlorophyll contents (**Figure 7B**), and lower Fv/Fm values (**Figure 7C**). These findings were further corroborated by the chlorophyll fluorescence imaging results (**Figure 7A**). These experimental results indicate that SlHSP17.3 contributes to enhance the salt resistance of transgenic *Arabidopsis* plants.

### 3.6 *SlHSP17.3* overexpression decreases the ROS content in transgenic *Arabidopsis*

**Figure 8:**
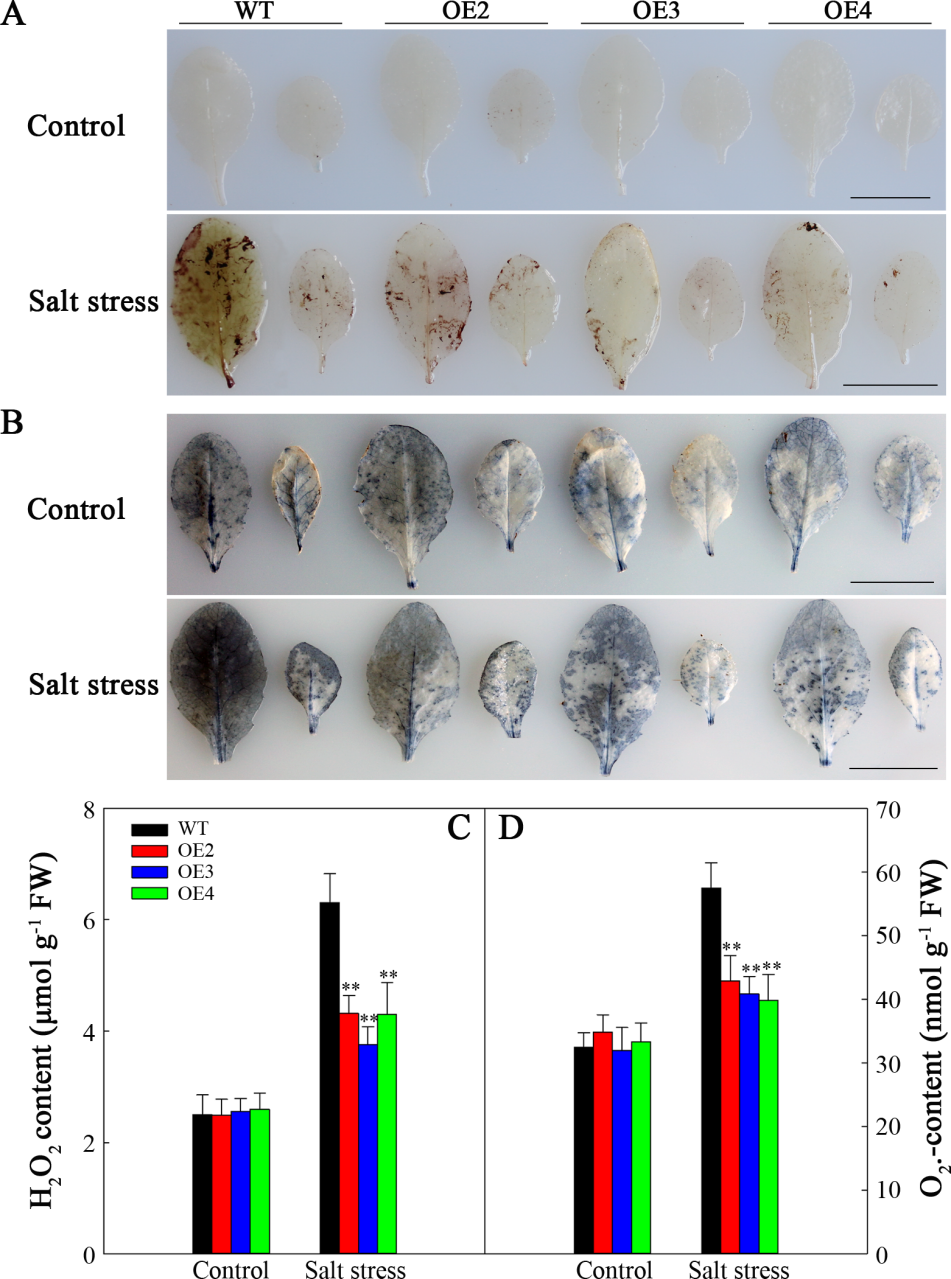
ROS correlation analysis of the WT and transgenic lines under salt stress. **(A)** DAB staining for H_2_O_2_. **(B)** NBT staining for O_2_^•−^. **(C)** H_2_O_2_ content. **(D)** O_2_^•−^ content. Scale bars = 1 cm. Significance levels are shown as ** for *p* < 0.01.

Salt stress typically leads to the generation of ROS. DAB and NBT staining were conducted to assess two primary ROS species, namely, H_2_O_2_ and O_2_^•−^, respectively. As shown in **Figure 8**, prior to treatment, the levels of H_2_O_2_ and O_2_^•−^ were relatively low, especially H_2_O_2_, and the differences in WT compared with transgenic plants were not significant. Nonetheless, after two-day salt stress, brown polymerization product (DAB staining) accumulation noticeably elevated, particularly in the WT (**Figure 8A**). Similar trends were noted for the O_2_^•−^ level (**Figure 8B**). The H_2_O_2_ and O_2_^•−^ levels were consistent (**Figures 8C, D**). Therefore, *SlHSP17.3* overexpression mitigates H_2_O_2_ and O_2_^•−^ accumulation.

**Figure 9:**
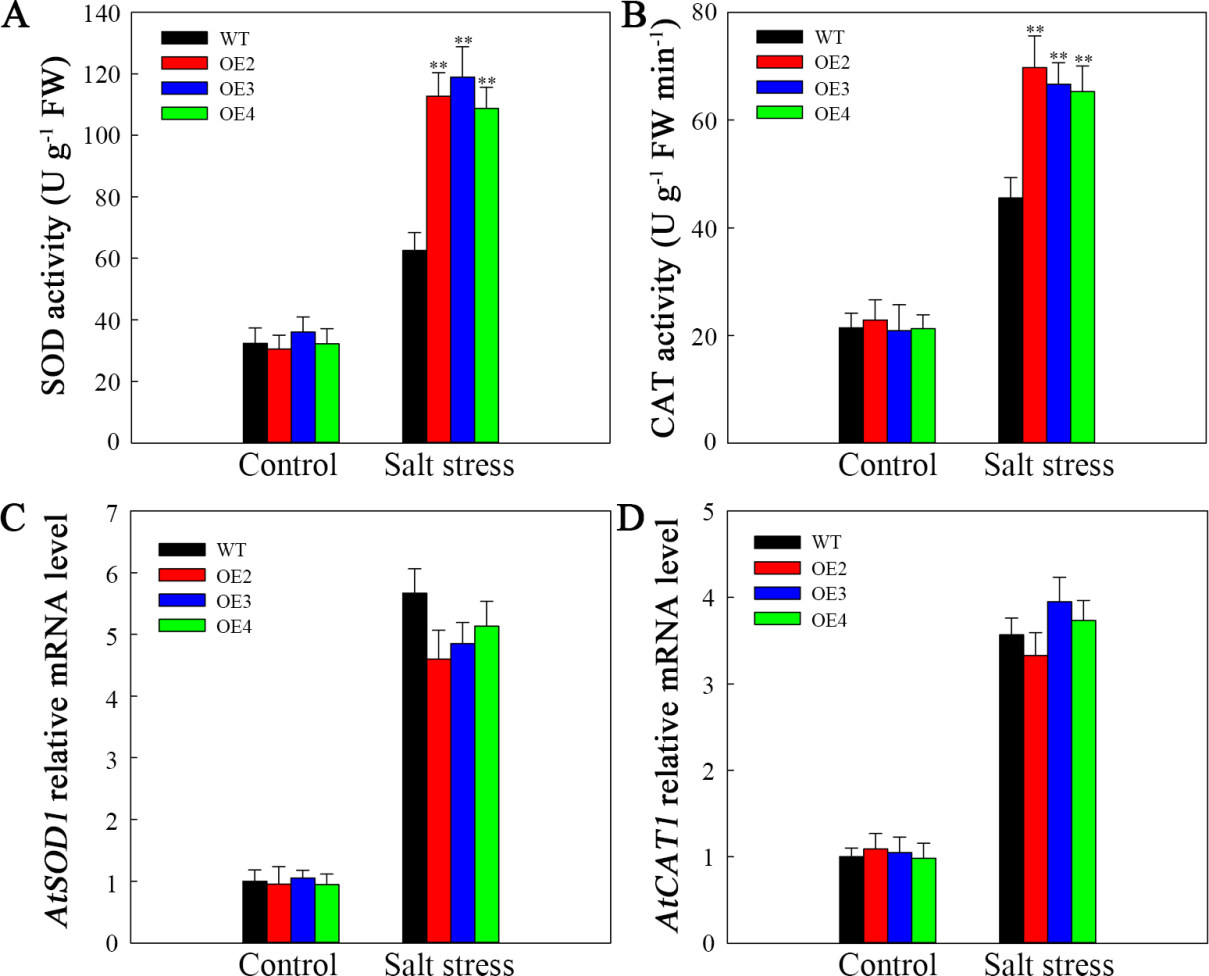
Antioxidase activities and antioxidase gene levels upon salt stress. **(A)** SOD activity. **(B)** CAT activity. **(C)** *AtSOD1* expression. Database accession number of *AtSOD1*: AT1G08830. **(D)** *AtCAT1* expression. Database accession number of *AtCAT1*: AT1G20630. Significance levels are shown as ** for *p* < 0.01.

The differences in SOD and CAT activities were of no statistical significance between the WT plants and the transgenic lines under normal conditions. Nevertheless, following two-day salt stress, the SOD and CAT activities increased to varying degrees. Nonetheless, compared to those in the WT lines, the increasing magnitudes of their activities within transgenic plants increased (**Figures 9A, B**). To investigate the reasons behind these alterations in enzyme activity, *AtSOD1* and *AtCAT1* expression was assessed via RT-qPCR. As shown in **Figures 9C, D**, *AtSOD1*, and *AtCAT1* expression was not markedly difference in transgenic plants compared with WT under normal conditions. At two days post salt stress, the expression of *AtSOD1* and *AtCAT1* significantly increased; however, there were no significant differences of the *AtSOD1* and *AtCAT1* expression level in WT and the transgenic lines. Therefore, the decreased ROS contents within the transgenic lines are associated with increased SOD and CAT activities attributed to *SlHSP17.3* overexpression.

### 3.7 *SlHSP17.3* overexpression alleviated salt-induced damage to cells

**Figure 10:**
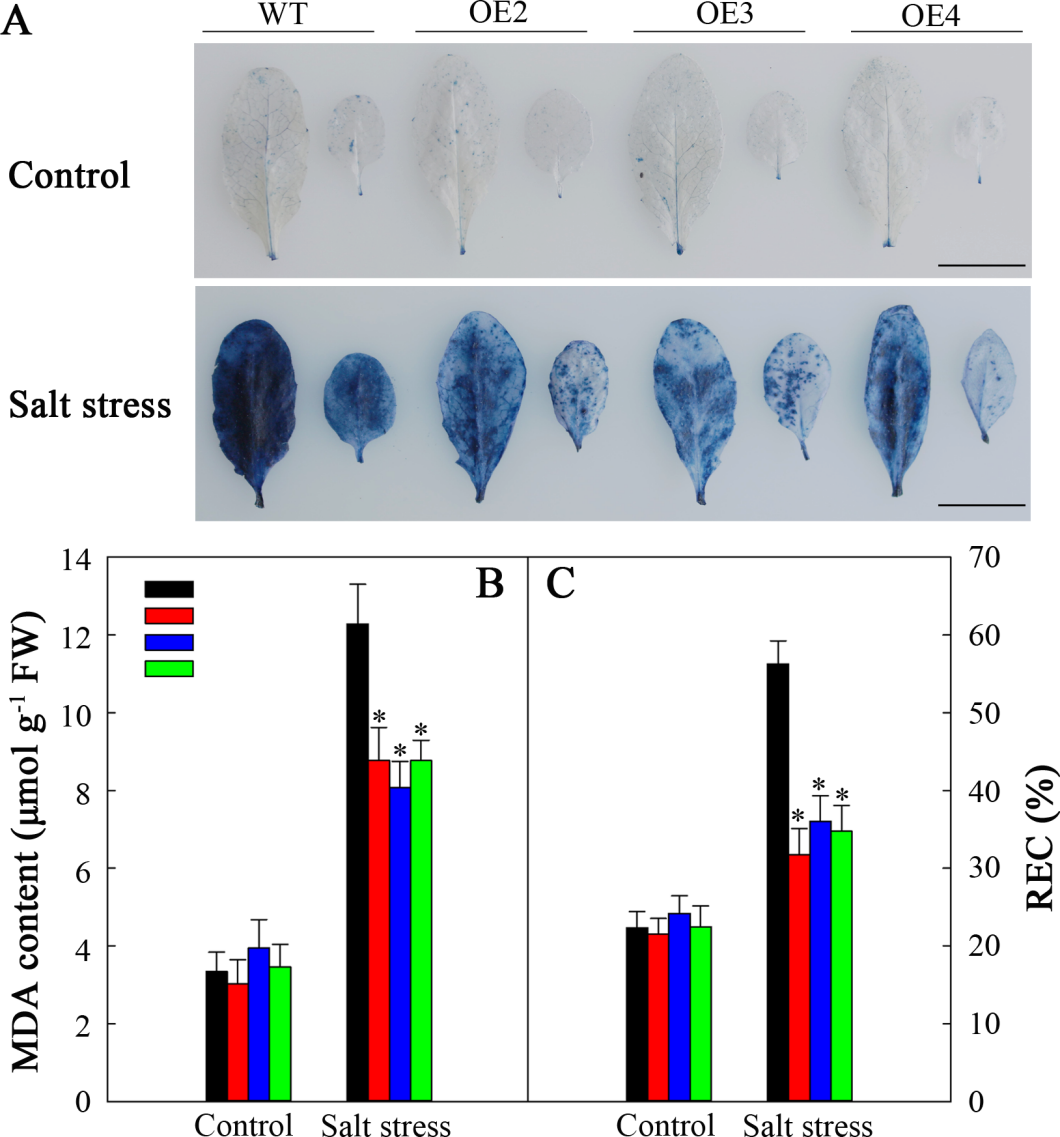
Cell injury in the WT and transgenic lines. **(A)** Trypan blue staining. The top panel shows plants under normal conditions, whereas the bottom panel shows plants under salt stress treatment for five days. **(B)** MDA levels of the WT and transgenic lines. **(C)** REC of the WT and transgenic lines. Scale bars = 1 cm. Significance levels are shown as * for *p* < 0.05.

The membranes of plant cells exhibit extremely high susceptibility to ROS-mediated lipid peroxidation. Trypan blue staining was used to analyze this susceptibility. After fiev days of salt stress, the WT plants presented darker blue coloration than did the transgenic plants (**Figure 10A**). Nonetheless, each plant had a similar degree of blue staining in the natural growth situations. To demonstrate the above findings, we assessed the MDA level and REC, which are known indicators of cell injury. Without salt stress, these indicators were not noticeably different between transgenic plants and WT. However, after salt stress, both indicators increased, with a more significant increase observed in WT than in those in the transgenic lines (**Figures 10B, C**). Consequently, *SlHSP17.3* overexpression provides protection against salt stress-induced cell injury.

### 3.8 *SlHSP17.3* overexpression promotes the expression of certain stress-related genes upon salt stress

**Figure 11:**
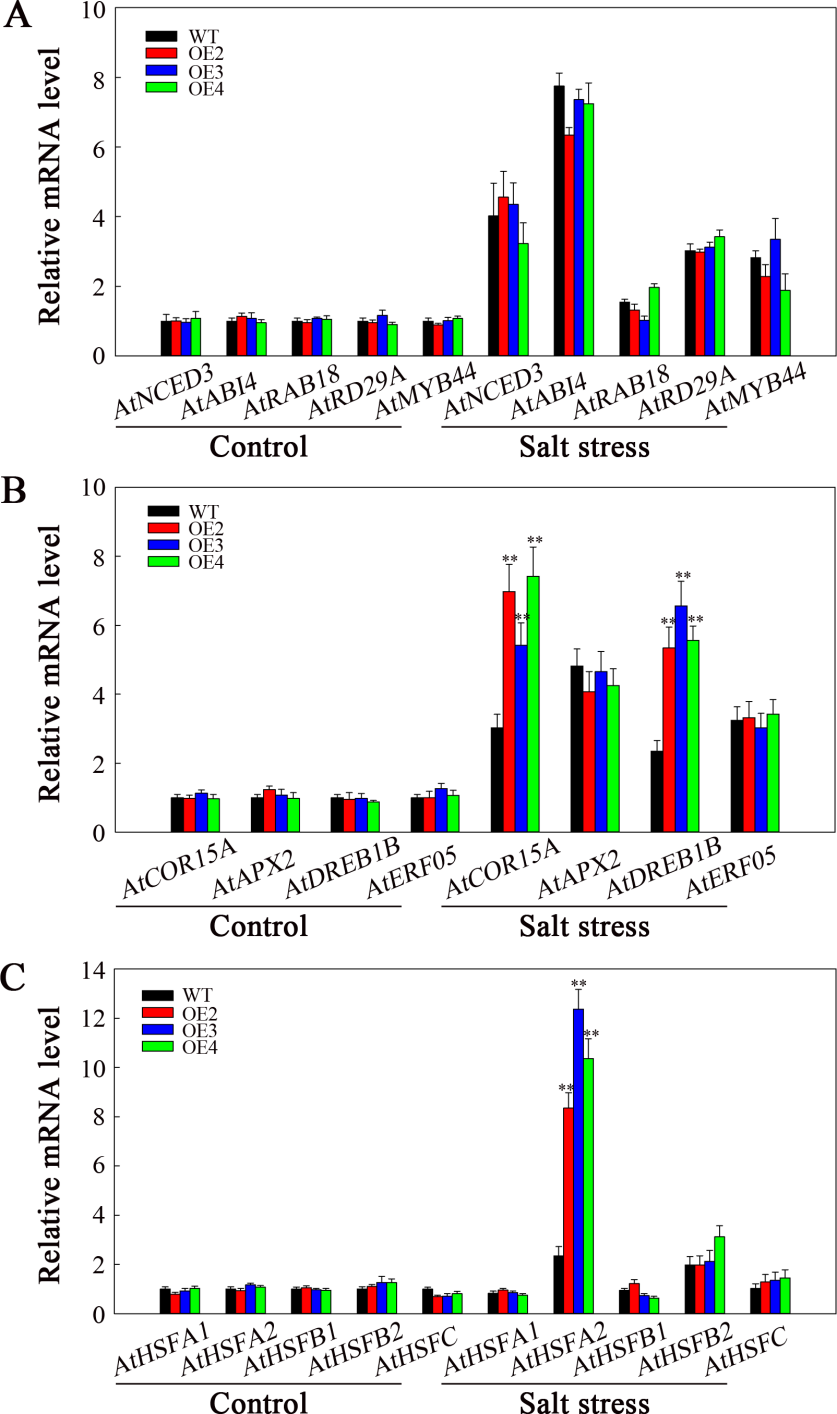
Analysis of ABA- and stress-related gene expression under salt stress. **(A)** ABA biosynthesis and signaling-related gene expression. **(B)** Stress-related gene expression. **(C)** *AtHSF* expression. Significance levels are shown as ** for *p* < 0.01.

To explore how SlHSP17.3 promotes the salt resistance of transgenic *Arabidopsis*, we carried out RT-qPCR to assess various factors related to ABA biosynthesis, signaling, and stress response. Before or after salt stress, the expression levels of genes related to ABA biosynthesis (*AtNCED3* and *AtABI4*) and ABA signaling (*AtRAB18*, *AtRD29A*, and *AtMYB44*) were not markedly different in WT and transgenic plants. However, following salt stress treatment, the expression of these genes increased in both the transgenic and WT plants (**Figure 11A**). Additionally, among the stress-related genes analyzed (*AtCOR15*, *AtAPX2*, *AtDREB1B*, and *AtERF05*), the expression of *AtCOR15* and *AtDREB1B* significantly increased among transgenic plants compared with WT following salt stress (**Figure 11B**). Moreover, considering that heat shock transcription factors respond to heat stress and are related to other stress responses, we examined the expression of several of these factors (*AtHSFA1*, *AtHSFA2*, *AtHSFB1*, *AtHSFB2*, and *AtHSFC*). After salt stress, *AtHSFA2* expression markedly increased in the transgenic plants compared with WT lines (**Figure 11C**). Consequently, such increased salt resistance in *SlHSP17.3*-overexpressing transgenic *Arabidopsis* plants could be associated with the upregulation of certain stress-related genes and certain HSFs.

## 4 Discussion

sHSPs constitute a group of molecular chaperones widely distributed in plants that provide protection against various environmental stresses. Many sHSPs are detected across various plants (Lopes-Caitar et al., 2013; Pandey et al., 2015; Tao et al., 2015; Guo et al., 2015). We have identified at least 42 sHSP proteins in tomato (unpublished). While recent studies on tomato sHSPs have focused mainly on their response to temperature stress, little attention has been given to understanding their effects on salt stress. For example, *MasHSP24.4* overexpression enhances the high-temperature tolerance of transgenic tomato plants (Mahesh et al., 2013), and specific sHSPs, such as SlHsp17.4-CII and SlHsp23.8-M, play direct roles in chilling tolerance mechanisms in tomato genotypes (Ré et al., 2017). Additionally, SlHSP17.7 has been suggested to act as a cofactor for SlCCX1-like proteins, maintaining intracellular Ca^2+^ homeostasis and reducing cold stress sensitivity (Zhang et al., 2020). We isolated a novel tomato *sHSP* gene (*SlHSP17.3*) and found that its expression was associated with salt stress (**Figure 4**). Consistent with previous research (Port et al., 2004), our analysis revealed that SlHSP17.3 contains a characteristic α-crystallin domain (ACD) with a β-folded structure (**Supplementary Figure S3**). Evolutionary analysis indicated that SlHSP17.3 belongs to the cytosolic class II sHSP protein group (**Figure 1**). Subcellular localization experiments confirmed its cytoplasmic localization (**Figure 2**). Furthermore, functional studies demonstrated the positive influence of SlHSP17.3 on salt stress in transgenic *Arabidopsis*.

Salt stress poses a significant challenge to modern agriculture, profoundly impacting plant development and crop production. Many studies have indicated that plant sHSP proteins can exert both positive and negative regulatory effects upon plants when responding to salt stress (Jiang et al., 2009; Sun et al., 2016; Wang et al., 2020; He et al., 2021; Qin et al., 2022). Our findings revealed that overexpressing *SlHSP17.3* promoted the salt resistance of transgenic *Arabidopsis* plants. After salt treatment, observations of the germination rate, seedling growth, membrane damage, mature transgenic plant phenotype, and associated physiological indices suggest that overexpressing *SlHSP17.3* enhances the salt resistance of transgenic *Arabidopsis* (**Figure 6**, **Figure 7**, **Figure 10**). Taken together, our findings indicate the significant impact of SlHSP17.3 on plant responses to salt stress.

Furthermore, our research highlights the crucial role of SlHSP17.3 in alleviating salt-induced oxidative stress. ROS accumulation is a common consequence of salt stress, leading to cell injury and dysfunction (Napieraj et al., 2020). Both NBT and DAB staining, along with quantification, clearly demonstrated that overexpressing *SlHSP17.3* substantially reduced ROS accumulation in salt-stressed transgenic *Arabidopsis* (**Figure 8**). This decrease in ROS levels is associated with increased antioxidase activities in *SlHSP17.3*-overexpressing plants, as shown in **Figure 9A**. Notably, this increased antioxidant enzyme activity does not correspond to elevated expression of antioxidant enzyme-encoding genes (**Figure 9B**). Instead, this finding suggests that SlHSP17.3 likely exerts its protective function as a molecular chaperone directly against oxidase activity. The observed upregulation of antioxidant enzymes and simultaneous reduction in ROS levels in *SlHSP17.3*-overexpressing plants upon salt stress underscore the pivotal role of SlHSP17.3 in scavenging ROS and mitigating oxidative stress. The above results provide insights into the intricate interplay between SlHSP17.3-mediated protein protection and ROS detoxification pathways, thereby enhancing overall stress resistance in plants.

sHSP protein expression in plants can positively or negatively impact salt stress by engaging in ABA-dependent/independent pathways, plant photosynthesis, and various stress response mechanisms (Sun et al., 2016; Li et al., 2018; Wang et al., 2020; He et al., 2021). Central to this regulatory network are the ABA responsiveness element (ABRE) and anaerobic response element (ARE) detected within *sHSP* gene promoters, which can be recognized by transcription factors, including AREB/ABF and MYB (Fujita et al., 2011; Banerjee and Roychoudhury, 2017). To explore the regulatory pathways of SlHSP17.3 under salt stress in greater depth, this study initially examined cis-acting elements within the *SlHSP17.3* promoter and identified both ABRE and ARE (**Supplementary Table S3**). Consequently, we treated plants with ABA to analyze *SlHSP17.3* gene expression, and our results revealed that the induction of *SlHSP17.3* gene expression by ABA was not significant (**Supplementary Figure S4**). Additionally, the ABA content in the leaves of four-week-old WT and transgenic plants was measured with and without salt stress treatment. Although the ABA content increased in all lines after salt stress treatment, there was no significant difference in the ABA content between the WT and transgenic plants (**Supplementary Figure S5**). On the basis of our results, SlHSP17.3 may have different functions than those reported in previous studies. Then, we used RT-qPCR to identify ABA biosynthesis-, signaling-, and stress-related gene levels under salt stress. Following salt stress treatment, genes related to ABA biosynthesis (*AtNCED3* and *AtABI4*) and ABA signaling (*AtRAB18*, *AtRD29A*, and *AtMYB44*) were upregulated to different extents. However, gene expression was not significantly different in transgenic plants compared with WT (**Figure 11A**). After salt stress, *AtCOR15* and *AtDREB1B* expression in transgenic plants markedly increased compared with that in WT (**Figure 11B**). Based on these results, *SlHSP17.3* overexpression enhances salt tolerance of transgenic *Arabidopsis* in an ABA-independent pathway through the up-regulation of certain stress-related genes. Furthermore, HSFs are related to heat shock responses and salt stress, where they collaborate with HSPs to improve plant resilience (Wang et al., 2019a; Iqbal et al., 2022). Therefore, selected HSF levels were examined, revealing that *AtHSFA2* was markedly up-regulated in transgenic plants relative to WT lines following salt stress (**Figure 11C**). According to the above findings, overexpression of *SlHSP17.3* contributes to the upregulation of specific *HSFs*, thereby increasing salt stress resistance in transgenic plants.

Overall, we separated and identified a novel sHSP protein (SlHSP17.3) in tomato that belongs to the cytosolic class II sHSP family. Overexpressing *SlHSP17.3* can alleviate ROS accumulation in salt-stressed transgenic *Arabidopsis* plants, and it can improve the salt stress resistance of transgenic *Arabidopsis* plants through up-regulating *AtCOR15*, *AtDREB1B*, and *AtHSFA2*.

## Data Availability

The original contributions presented in the study are included in the article/**Supplementary Material**, further inquiries can be directed to the corresponding author/s.
