# Supplementary material for: A small heat shock protein (SlHSP17.3) in tomato plays a positive role in salt stress
Source: Front Plant Sci. 2024 Oct 11;15:1443625. doi: 10.3389/fpls.2024.1443625 (PMC11503465; doi:10.3389/fpls.2024.1443625)
Supplement: Supplementary file 1 [file DataSheet1.docx]

Supplementary Material

**Supplementary Table 1.** The primers used for the RT-qPCR reactions.

| Gene name | Accession No. | Sequence (5′-3′) |
| --- | --- | --- |
| *Actin1* | LOC544055 | *EF1α*-F, GGAACTTGAGAAGGAGCCTAAG  *EF1α*-R, CAACACCAACAGCAACAGTCT |
| *Actin2* | AT4G05320 | *AtUbiquitin* -F, CGGAAAGCAGTTGGAGGATGG  *AtUbiquitin*-R, CGGAGCCTGAGAACAAGATGAAG |
| *SlHSP17.3* | SGNU579132 | *SlHSP17.3*-F, AGCTGCTGGTGAAGATTCCGT  *SlHSP17.3*-R, AGCACATTGTCTTCTTCTACCT |
| *AtSOD1* | AT1G08830 | *AtSOD1* -F, ATGCTAATCGACATGCTGGTG  *AtSOD1* -R, AGTAGCCAGGCTGAGTTCAT |
| *AtCAT1* | AT1G20630 | *AtCAT1*-F, TGAAATCCTATAAACTCAATATGCTC  *AtCAT1*-R, AACAGGAACTAGTACCCTTCTTTAAGC |
| *AtNCED3* | AT3G14440 | *AtNCED3*-F, GCCGAAGATTCATCGAACAT  *AtNCED3*-R, CGAGTTGATTCACCGGTTTT |
| *AtABI4* | AT2G40220 | *AtABI4*-F, TCAATAACTCATCCACCGC  *AtABI4*-R, AAAATCCCAAATACTCCCC |
| *AtRAB18* | AT5G66400 | *AtRAB18* -F, GTCTTACTGCTGAAGGTTCGTCTG  *AtRAB18* -R, ATCCAAGATGCTGCGGTTTAGG |
| *AtRD29A* | AT5G52310 | *AtRD29A*-F, GTGACGACGAAGTTACCTATCTCC  *AtRD29A*-R, TCTCCGCCACATAATCTCTACCC |
| *AtMYB44* | AT5G67300 | *AtMYB44*-F, GTGGATGAAGCGTGTGG  *AtMYB44*-R, GAAAGAAGGGCGTGTGT |
| *AtCOR15A* | AT2G42540 | *AtCOR15A*-F, TGGCGATGTCTTTCTCAGGA  *AtCOR15A*-R, AGGATGTTGCCGTCACCTTT |
| *AtAPX2* | AT3G09640 | *AtAPX2*-F, AGTTGCTGTTGAGATCACTG  *AtAPX2*-R, GAACGCTCCTTGTGGCACCG |
| *AtDREB1B* | AT4G25490 | *AtDREB1B*-F, AGCCAGCCAACCACACAG  *AtDREB1B*-R, TAGCGAAGCAATCCCACG |
| *AtERF05* | AT5G47230 | *AtERF05*-F, TGAAGACGGAACAGAGCG  *AtERF05*-R, GGAGATAACGGCGACAGA |
| *AtHSFA1* | AT4G17750 | *AtHSFA1*-F, ACGGGTTCTCATCTCCAAAC  *AtHSFA1*-R, ATCTGGGAAGTCTCTGGCAT |
| *AtHSFA2* | AT2G26150 | *AtHSFA2*-F, ACATGGGTTTGCAGAATGTG  *AtHSFA2*-R, GCAACTTGACTCTTGGAGCT |
| *AtHSFB1* | AT4G36990 | *AtHSFB1*-F, AGCTCCACGTCATCACCCGGT  *AtHSFB1*-R, GGTCTTACTTTCAGATGACCCG |
| *AtHSFB2* | AT5G62020 | *AtHSFB2*-F, ACCTCGTTGAAGATAGTTCC  *AtHSFB2*-R, ATCCGTAAGTGTTGAGCTGACG |
| *AtHSFC* | AT3G24520 | *AtHSFC* -F, ACCTCGTTGAAGATAGTTCC  *AtHSFC* -R, ATCCTCTGAATCTCTGCCTCT |

**Supplementary Table 2.** Prediction of subcellular localization of SlHSP17.3.

| Location weight | LocDB | PotLocDB | Neural Nets | Pentamers | Integral |
| --- | --- | --- | --- | --- | --- |
| Nuclear | 2.8 | 0.0 | 0.00 | 0.00 | 1.93 |
| Plasma membrane | 0.0 | 0.0 | 0.96 | 0.00 | 0.21 |
| Extracellular | 0.0 | 0.0 | 0.96 | 0.14 | 0.00 |
| Cytoplasmic | 4.8 | 1.6 | 0.00 | 4.35 | 4.26 |
| Mitochondrial | 0.0 | 0.0 | 0.00 | 1.45 | 0.02 |
| Endoplasm.retic. | 0.0 | 0.7 | 0.00 | 0.08 | 0.68 |
| Peroxisomal | 2.4 | 0.6 | 0.96 | 0.00 | 2.38 |
| Golgi | 0.0 | 0.0 | 0.11 | 0.48 | 0.00 |
| Chloroplast | 0.0 | 0.0 | 0.00 | 0.00 | 0.30 |
| Vacuolar | 0.0 | 0.0 | 0.00 | 0.00 | 0.21 |

The data have been obtained with ProtComp 9.0 (http://linux1.softberry.com/berry.phtml). Integral prediction of SlHSP17.3 protein location: Cytoplasmic with score 4.26.

**Supplementary Table 3.** Main putative *cis*-acting elements and their positions in the 2000 bp upstream the *SlHSP17.3* transcription initiation site.

| *Cis*-element | Consensus sequence | Responsiveness | Position |
| --- | --- | --- | --- |
| ABRE | ACGTG | ABA responsiveness | -1795 |
| ARE | AAACCA | anaerobic response | -1113 |
| TCA-element | CCATCTTTTT | salicylic acid | -357, -1438 |
| CGTCA-motif | CGTCA | MeJA | -1085 |
| LTR | CCGAAA | low temperature | -595, -1884 |
| MBS | CAACTG | drought | -1060 |
| MRE | AACCTAA | light | -472 |

The data have been obtained with PlantCARE (<http://bioinformatics.psb.ugent.be/webtools/plantcare/html/>). Indicated positions are relative to the transcription initiation site.


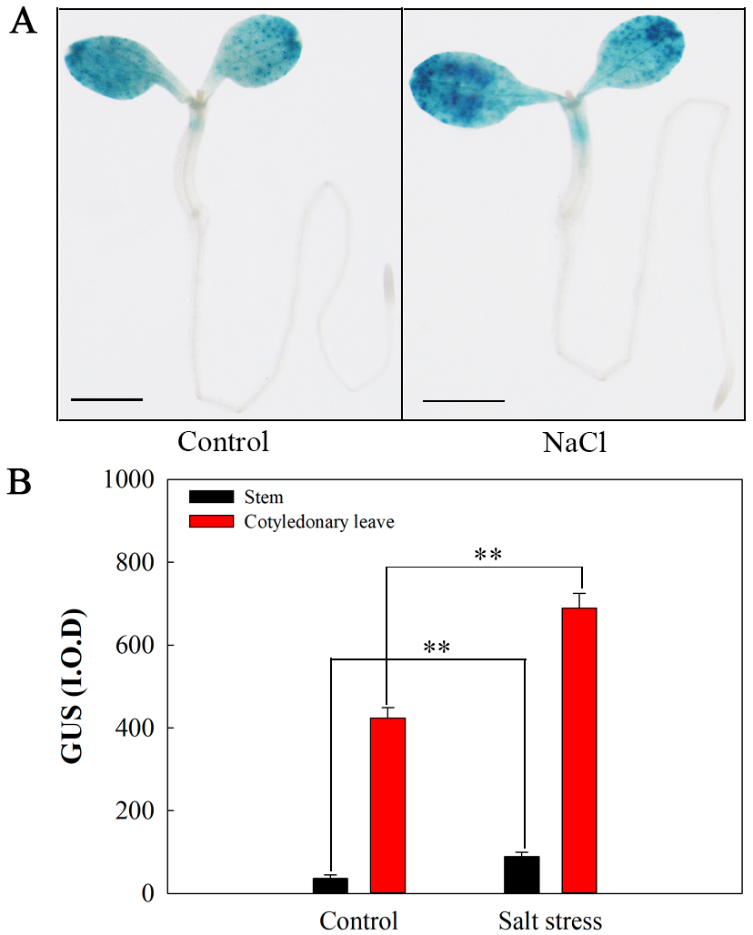


**Supplementary Figure 1.** GUS staining analysis of 3-day-old seedlings under normal conditions and after exposure to salt stress for 1 day. (A) GUS staining. Scale bars=1 mm. (B) Quantitative analysis of GUS staining for (A). Significance levels are shown to be ** for *p* < 0.01.


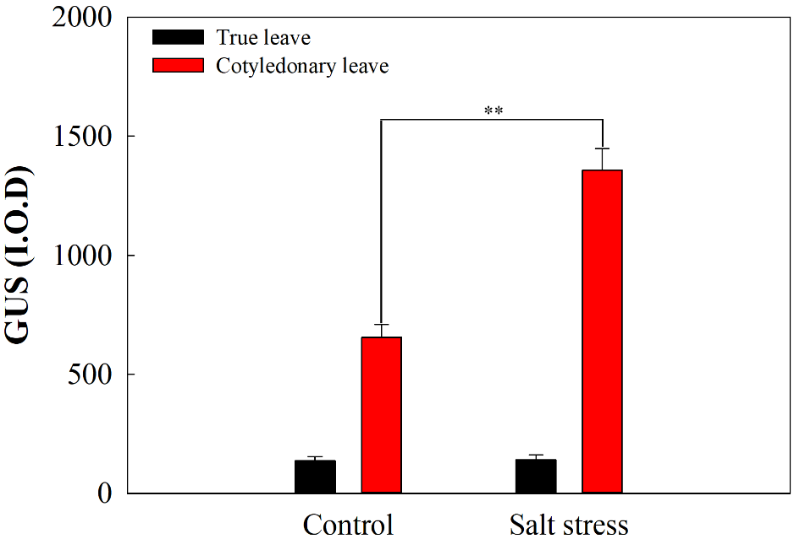


**Supplementary Figure 2.** Quantitative analysis of GUS staining in true leaves and cotyledonary leaves for figure 4B. Significance levels are shown to be ** for *p* < 0.01.


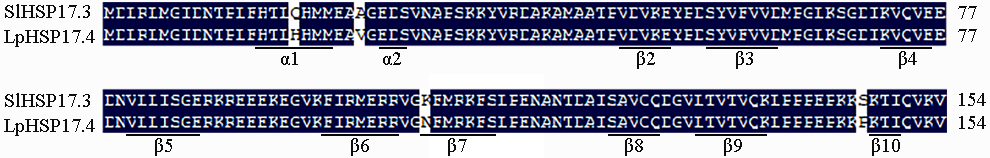


**Supplementary Figure 3.** Analysis of the amino acid sequence of SlHSP17.3 protein. Secondary structure prediction shows α-helix at the N-terminus and β-sheets in the C-terminal ACD (β2-β9) underlined. Database accession number of LpHSP17.4: AY608694.


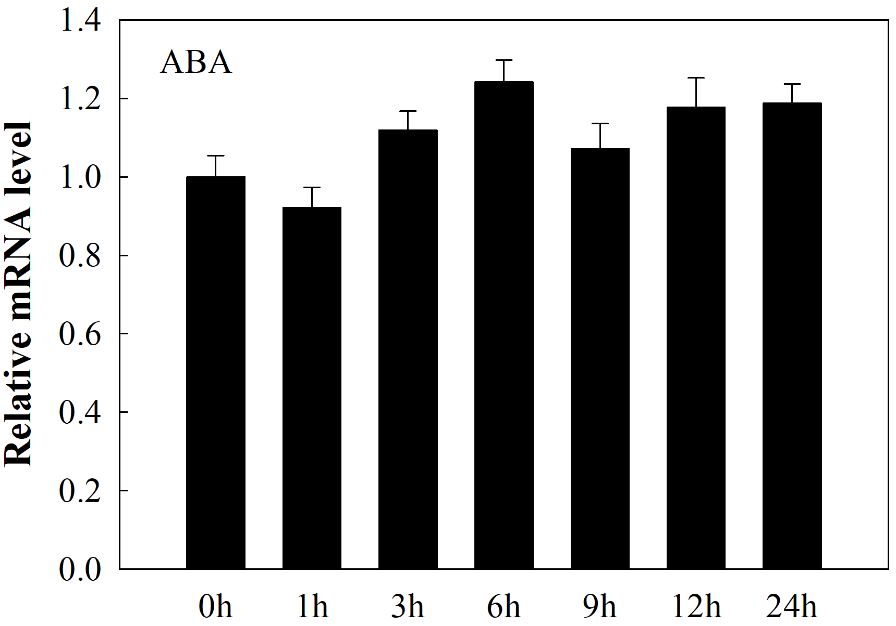


**Supplementary Figure 4.** Analysis of *SlHSP17.3* expression induced by ABA.


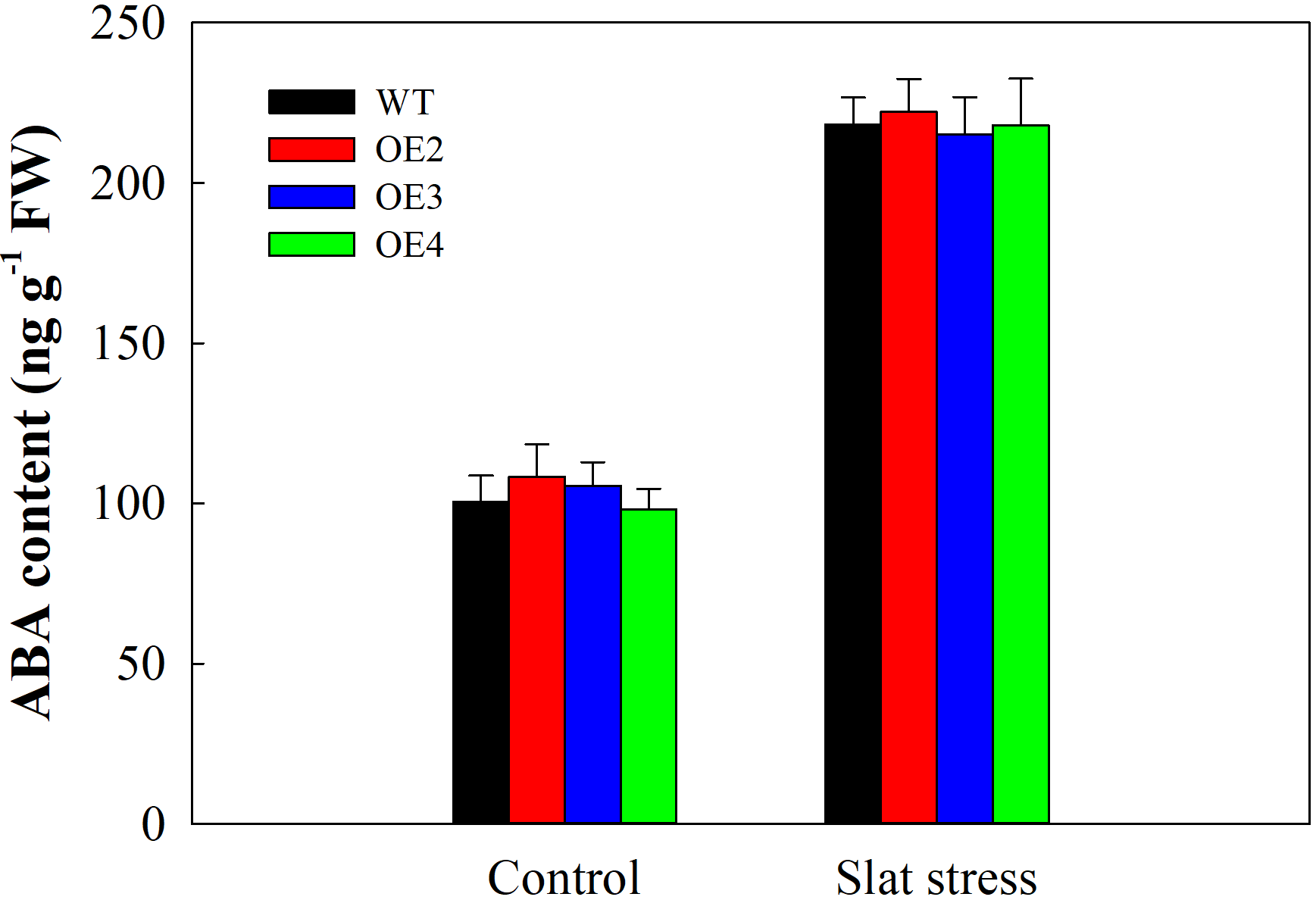


**Supplementary Figure 5.** The ABA content in leaves of 4-week-old WT and transgenic plants.
